# *In silico *pathway reconstruction: Iron-sulfur cluster biogenesis in *Saccharomyces cerevisiae*

**DOI:** 10.1186/1752-0509-1-10

**Published:** 2007-01-31

**Authors:** Rui Alves, Albert Sorribas

**Affiliations:** 1 Departament de Ciencies Mediques Basiques, Universidad de Lleida, Montserrat Roig 2, 25008 Lleida, Spain

## Abstract

**Background:**

Current advances in genomics, proteomics and other areas of molecular biology make the identification and reconstruction of novel pathways an emerging area of great interest. One such class of pathways is involved in the biogenesis of Iron-Sulfur Clusters (ISC).

**Results:**

Our goal is the development of a new approach based on the use and combination of mathematical, theoretical and computational methods to identify the topology of a target network. In this approach, mathematical models play a central role for the evaluation of the alternative network structures that arise from literature data-mining, phylogenetic profiling, structural methods, and human curation. As a test case, we reconstruct the topology of the reaction and regulatory network for the mitochondrial ISC biogenesis pathway in *S. cerevisiae*. Predictions regarding how proteins act in ISC biogenesis are validated by comparison with published experimental results. For example, the predicted role of Arh1 and Yah1 and some of the interactions we predict for Grx5 both matches experimental evidence. A putative role for frataxin in directly regulating mitochondrial iron import is discarded from our analysis, which agrees with also published experimental results. Additionally, we propose a number of experiments for testing other predictions and further improve the identification of the network structure.

**Conclusion:**

We propose and apply an iterative *in silico *procedure for predictive reconstruction of the network topology of metabolic pathways. The procedure combines structural bioinformatics tools and mathematical modeling techniques that allow the reconstruction of biochemical networks. Using the Iron Sulfur cluster biogenesis in *S. cerevisiae *as a test case we indicate how this procedure can be used to analyze and validate the network model against experimental results. Critical evaluation of the obtained results through this procedure allows devising new wet lab experiments to confirm its predictions or provide alternative explanations for further improving the models.

## Background

Increasing amounts of data that can be mined for information about how proteins in cells assemble as metabolic pathways, signal transduction pathways, and gene circuits, are generated each day. Datasets available for such tasks include the primary literature, large scale micro array experiments, whole genome two hybrid screenings, full genome sequences, and the patterns of conserved/non-conserved homologues and orthologues in them. Theoretical and computational methods are being developed and used to analyze these different types of data and infer networks of proteins or genes that are involved in the same cellular process(es) (e.g. [1-10]).

In general, the networks derived by the computational analysis of these data are static, in the sense that they provide little information, if any, about the flow of causality and events in the process and no information about the dynamics of the processes and its regulation (however, see [11]). For example, the involvement of proteins X, Y and Z in a process does not elucidate if X catalyzes a reaction that produces a substrate for another reaction catalyzed by Z or by Y, or if X modulates Y or Z activity. This can be an important problem while assembling the network structure of either novel pathways (e.g. Iron-Sulfur Cluster biogenesis) or complex pathways with an unclear reaction and regulation network, (e. g. cell cycle). Thus, it is a challenge to transform the network of interactions inferred from the analysis of static data into a causal network that allows for the creation of mathematical models whose dynamic behavior can be analyzed and tested against experimental observations.

To achieve such a goal, strategies that combine the different theoretical and computational methods to identify proteins and generate a set of plausible alternative network topologies for the process of interest are needed. Such networks can then be translated into mathematical models whose dynamic behavior can be analyzed and compared to that of the real system, thus discriminating against some of the proposed topologies when they do not reproduce the expected behavior. Such an analytical process integrates *omics *data and provides testable predictions and information about systemic behavior.

The more than likely absence of known mechanistic and kinetic data for each of the individual proteins in a novel pathway hinders the process of translating network topology into a mathematical model. A way around the problem is by using approximation theory [12]. This well-established methodology approximates the continuous functions that typically describe the kinetics of protein processes by using, for example, truncated Taylor series, either in linear or non-linear spaces (see e.g. [13-19]). Among the non-linear approximations, the power-law formalism provides a useful representation that comes associated with powerful and eclectic analytical methods (see e.g. [20-24]).

In this paper, we shall focus on defining and applying a global strategy combining bioinformatics tools and mathematical modeling to reconstruct the network structure of a pathway. Computational tools will be used for a) obtaining relevant information on genes and proteins that are identified as playing a role in the target pathway, b) checking putative interactions between proteins, c) testing the co-evolution of different proteins, and d) for setting-up alternative networks that accommodate all this information. Then, expert knowledge is used to curate the set of alternative network structures. Finally, mathematical models are used to explore the systemic behavior of each alternative network and comparing it with existing experimental data.

As a benchmark problem we shall focus on the Iron-Sulfur Cluster (ISC) biogenesis pathway. ISC are widespread cofactors of proteins that work as catalytic mediators, as electron transport mediators, and as sensors for the oxidation state of the cell and of its environment [25-32]. Although ISC have been known to assemble autonomously in proteins, in recent years, an evolutionarily conserved set of proteins that controls this assembly has been identified [29,33,34]. In eukaryotes, initial ISC biogenesis is mitochondrial [35]. Deregulation of ISC biogenesis in humans can create different pathological effects, leading to diseases such as Friedreich's ataxia, X-linked sideroblastic anemia, or hypochromic anemia. In yeast, deleting one ISC biogenesis gene creates cells that accumulate iron and have a decrease/deregulation in the activity of ISC dependent proteins. The extent of the phenotype ranges from mild (e.g. Δ*GRX5 *strains [36]) to lethal (e.g. Δ*ARH1 *strains [37]), depending on the protein that is mutated. Friedreich's ataxia is linked to mutations in one of the ISC biogenesis proteins (Frataxin) which has as a homologous protein in yeast the proteinYfh1 (Yeast Frataxin Homologue 1). Additionally, iron accumulation can lead to cellular aging and its associated diseases.

Although spontaneous assembly of ISC has been known to occur both *in vivo *and *in vitro*, it has been observed that mutations in a set of proteins that are evolutionarily conserved cause defects in ISC biogenesis. These proteins are evolutionarily conserved and form a putative ISC biogenesis pathway. The details and topology of this pathway are still not fully understood. In *S. cerevisiae*, the eukaryotic organism in which the ISC biogenesis has been more extensively studied, the following proteins are involved: Arh1, Yah1, Yfh1, Isu1, Isu2, Isa1, Isa2, Nfu1, Nfs1, Isd11, Mge1, Ssq1, Jac1, Atm1 and Grx5 (Table 1). The current dogma in the field assumes that Isu1, Isu2, Isa1, Isa2 and Nfu1 are somehow the scaffolds where the ISC initially assembles before being transferred to the appropriate ISC dependent apo-proteins. However, recent results may be casting some doubt into this, as there appears to be some involvement of Isa1/Isa2 in Fe supply for the clusters of specific ISC dependent apo-proteins. Furthermore, the role of Nfu1 is unclear. Atm1 is likely to be the transporter involved in exporting ISC to the cytoplasm. Arh1 and Yah1 are a feredoxin reductase-feredoxin pair that probably regulates electron transfer during the initial assembly of the cluster. Nfs1 is a cysteine desulfurase that provides the sulfur for the clusters and Isd11 is fundamental for Nfs1 to fulfill its role. It is unclear how Isd11 facilitates the functions of Nfs1. In bacteria, some cysteine desulfurases also have an assistant protein that facilitates the transfer of sulfur to the clusters via formation of and S-S bond. However, Isd11 does not have cysteine residues, which precludes such a mechanism for its action. Ssq1 (HSP 70 like protein), Jac1 (HSP 40 like protein) and Mge1 (Nucleotide exchange factor) are protein chaperones that are involved in assisting the pathway, although their exact role is unclear. It has been shown that Isu1 activates the ATPase activity of the HSP70 type chaperone Ssq1. Atm1 appears to participate in the exporting of the ISC clusters from the mitochondrial matrix to the cytoplasm. Again, the exact substrate of Atm1 is unknown. Grx5 is a monothyolic glutaredoxin whose function in ISC biogenesis is unclear. In prokaryotes this biogenesis is cytoplasmatic. In some cases more than one system is involved in the biogenesis of ISC. For example in *E. coli*, the ISC system (homologue to that of *S. cerevisiae*) [38-42] and the Suf system [43] are parallel systems that are involved in the biosynthesis of ISC. While the ISC system is the one responsible for regular assembly of ISC, the Suf system becomes important when the bacteria are under oxidative stress.

As mentioned in the previous paragraph, there is enough information to attribute a function to some of the proteins involved in ISC biogenesis in *S. cerevisiae*. This is the case for example of Nfs1, Isu1, Isu2, or Atm1. However, the role of other proteins is still not clear. For example, what do Isa1, Isa2 or Nfu1 do in the process? What is the role of the chaperones Ssq1-Jac1-Mge1 or of Grx5 in ISC biogenesis? Thus ISC biogenesis is a good benchmark problem for the application of the methodology we describe, as it will provide the chance to validate some of the prediction with published experimental results. Simultaneously, the methodology will generate biological insight regarding some of the proteins with an unclear role, thus creating an added value from the methodological and from the biological point of view. In previous papers [34,44,45] we combined structural bioinformatics with experiments and kinetic modeling to investigate the possible role of proteins Arh1, Yah1 and Grx5 in mitochondrial ISC biogenesis. In this paper we present a structured computational approach that is used to infer and analyze probable topologies for the global network of mitochondrial ISC biogenesis. We analyze seven of the proteins involved in the process (Arh1, Yah1, Yfh1, Grx5, Nfs1, Ssq1 and Jac1), proposing likely systemic roles for their action in ISC biogenesis.

## Results

The proteins that are known to be involved in ISC mitochondrial biogenesis in *S. cerevisiae *are shown in Table 1. We shall first describe how the different large scale datasets are analyzed and combined, using different computational tools in order to infer initial alternatives for the network assembly of the pathway. Then, we use mathematical modeling strategies to further analyze the different alternative and identify the most reliable network structure, based on comparing the dynamic behavior of the models to experimental observations.

### Pathway Reconstruction using automated literature analysis

**Figure 1:**
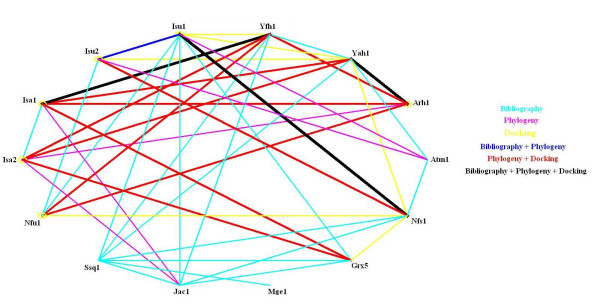
Network of protein and gene interactions as derived from different theoretical and computational approaches. Light blue lines connect genes that co-occur in papers, as determined using iHOP. Mauve lines connect genes that have a significant phylogenetic coincidence, as measured by both the mutual information and the transformed Hamming distance index we use. Yellow lines connect proteins that have either been experimentally reported to interact or have been predicted in this work via in silico protein docking as being more likely to interact. Dark blue lines connect genes that co-occur in the literature and have a significant Phylogenetic coincidence. Green lines connect genes/proteins that co-occur in the literature and have been predicted to interact by our in silico docking experiments. Red lines connect genes/proteins that have a significant Phylogenetic coincidence and are predicted to interact by our docking experiments. Black lines connect genes/proteins that co-occur in the literature, have a significant Phylogenetic conservation and have been predicted to interact by the docking experiments.

Automated bibliomic analysis of published abstracts and papers can be used to identify the different proteins or genes involved in a given process [46]. We have used the genes known to be involved in mitochondrial ISC biogenesis in *S. cerevisiae *to test this assumption. **iHOP **[47-49] is a Web-based tool that allows a fairly automated analysis of abstracts in search for gene names, generating a network of genes that are co-present in papers. This network, one expects, translates into a group of genes involved in the same process as our original gene of interest. When applied to the ISC biogenesis, iHOP identified all genes corresponding to the proteins that are thought to be involved in the process as well as some additional ones that are marginally important. The identification of the genes involved in the network was done by starting a new search for each of the gene names from Table 1. As a new gene was found during the search we analyzed the content of the abstract. If the abstract referred to ISC biogenesis or Fe metabolism and the gene was native to *S. cerevisiae*, the gene was added to the network model; otherwise it was not. If the new gene was added, then we appended its name to the original list of genes and used it as an initial seed in one of the searches. We continued this process manually until the results converged and no new gene name was added to the network. For each search, all genes known to be involved in ISC were found by iHOP. This suggests that semi-automated literature analysis methods are indeed effective in identifying known component parts of cellular processes of interest and in providing an initial network of proteins for the study. Figure 1 shows the network of interactions for the proteins involved in mitochondrial ISC biogenesis in *S. cerevisiae *as derived from the automated literature analysis. We have not included Isd11, as this gene works complementarily with Nfs1 [50,51].

### Pathway Reconstruction using Phylogenetic profiling

It is not possible to infer probable network structure(s) for the ISC biogenesis pathway just from the co-occurrence of genes in papers. Additional data is necessary in order to translate the group of proteins that are identified to be involved in the biological process of interest into a structured network of causal and regulatory relationships. Phylogenetic profiling [52,53] of the genes assists in adding some structure to the network. In this type of analysis, one searches for patterns of co-occurrence of sets of orthologues over a large number of fully sequenced genomes. The assumption behind the use of this approach is that, if the pattern of presence and absence of two or more proteins in a number of genomes is very close, this is an indication of co-evolution among the proteins. Such co-evolution can be taken as an indication that they are likely to be involved in the same cellular process(es).

Results from phylogenetic co-evolution analysis of the different genes involved in ISC biogenesis are summarized in Figure 1. This additional information already assists in inferring some form of causality to the network structure of the pathway. For example, Grx5 homologues coincide with Isa1 and Isa2 homologues in more genomes than with any other of the identified proteins. This suggests that Grx5 is more likely to act directly on these proteins than with other proteins that are also involved in ISC biogenesis. As can be seen in Table 1, Grx5 is a glutaredoxin that is likely to regulate (de)glutathionylation of protein cysteine residues. The phylogenetic analysis suggests that the scaffold proteins are likely targets for that regulation. Similarly, Atm1 homologues coincide with Isu1 and Isu2 homologues in more genomes than with homologues of other ISC biogenesis proteins. Because Atm1 is a membrane transporter, this result suggests that Isu1 and Isu2 are more likely to be involved in ISC transfer to the cytoplasm than other scaffold proteins. Sqq1, Jac1 and Mge1 are a chaperone, a co-chaperone, and a nucleotide exchange factor respectively. Homologues of the three proteins are present together in many genomes. Finally, Yah1 and Yfh1 homologues coincide in a high number of genomes, suggesting a common role for these two proteins.

### Pathway Reconstruction using low definition protein docking and in vitro protein interaction data

Alternatives for a partial network structure of the ISC biogenesis pathway have emerged from the phylogenetic analysis in the previous section. Additional information regarding this structure can be obtained through the use of protein docking methods [54-60].

Using protein docking to determine how proteins assemble in a pathway is not a standard procedure and may present various types of problems. First, proteins in the same pathway need not physically interact. Products from an early step may be released into solution and used by a protein involved in a later step of the process. However, this problem is not relevant in the current case. Because ISC are labile, a direct physical interaction between proteins is required for synthesis and transfer of the clusters. Second, protein modeling and/or docking is computationally very expensive. However, in the current case a limited number of proteins are involved in the target pathway. Thus, this problem is reduced to manageable levels. Third, docking any two proteins will always give a result. For example, consider proteins A and B that co-occur in the same cellular compartment and are involved in the same process, while protein C is located in another cellular compartment and has no role in the process of interest. If we dock protein A to protein B and then dock protein A to protein C, the score of the later docking may be much larger than that of the former. Choosing A and C as the preferred docking partners would be an erroneous result because A and C never meet *in vivo*. This problem is avoided in the present case because the proteins we are docking co-exist in the same cellular compartment and are involved in the same pathway. Fourth, docking a large aggregate of proteins that works together to form a pathway is still beyond the scope of protein docking. This is an important problem but we have made the assumption that, because physical interaction between proteins that catalyze a step is required, the individual docking results would be indicative of actual physical interactions.

*In silico *protein docking methods require protein structures. Of the proteins involved in ISC biogenesis in *S. cerevisiae *only Yfh1 has had its structure experimentally determined [61]. We built models for the structures of the remaining proteins. These models were obtained by homology modeling as described in the methods section.

Extending our previous work with this system [34,44,45], we use GRAMM [54-60] and Hex [62] to perform all against all *in silico *protein docking studies. The GRAMM methodology is specially suited to dock protein models because it averages over possible errors in the conformation that are unquestionably larger than in the case of crystallographic structures [55,63,64]. Figure 1 shows the four highest scores of interaction for each of the relevant genes. Several possibilities for the structure of the network can be inferred from these results. On one hand, both Nfs1 and Yfh1 show stronger docking scores with the four scaffold proteins. This suggests that both proteins act in the processes that assemble the cluster in the scaffolds. Arh1 and Yah1 are likely to be a pair that acts together, given that their mutual docking score is high. Grx5 docking scores are higher for Nfs1 and Arh1, suggesting that the later proteins are targets for the regulation of cysteine (de)glutathionylation by Grx5.

We have complemented the network of interactions inferred from the docking experiments with the interactions reported in two-hybrid screenings of yeast proteins from the published literature [34,65-69]. Within these data sets, we have only found evidence for interaction between Nfs1 and Isu1, Nfs1 and Isu2, Isa1 and Isa2, and between Isa1 and Grx5. The Nfs1-scaffold interactions are predicted by protein docking, thus suggesting that using docking methods to resolve pathway structure might be an adequate method, under similar circumstances. The Grx5-Isa1 interaction is consistent with the phylogenetic analysis reported in a previous section.

### Generating a curated pathway model using expert knowledge

The previous analysis suggests a skeletal structure for the ISC Biogenesis pathway. However, this structure needs to be further refined before it can be analyzed using mathematical modeling. A more detailed topology requires a deeper analysis of the data available in the literature. Additionally, non-genetic regulatory influences to the pathway are hard to identify automatically, and one must include such information using expert knowledge. At this stage, human curation is needed to turn the automated analysis of the previous sections into a network of reactions and to add further detail to the reaction topology and regulation. This curation is described in detail in the Supplementary Appendix [see Additional File 1].

### Alternative Mathematical Models

**Figure 2:**
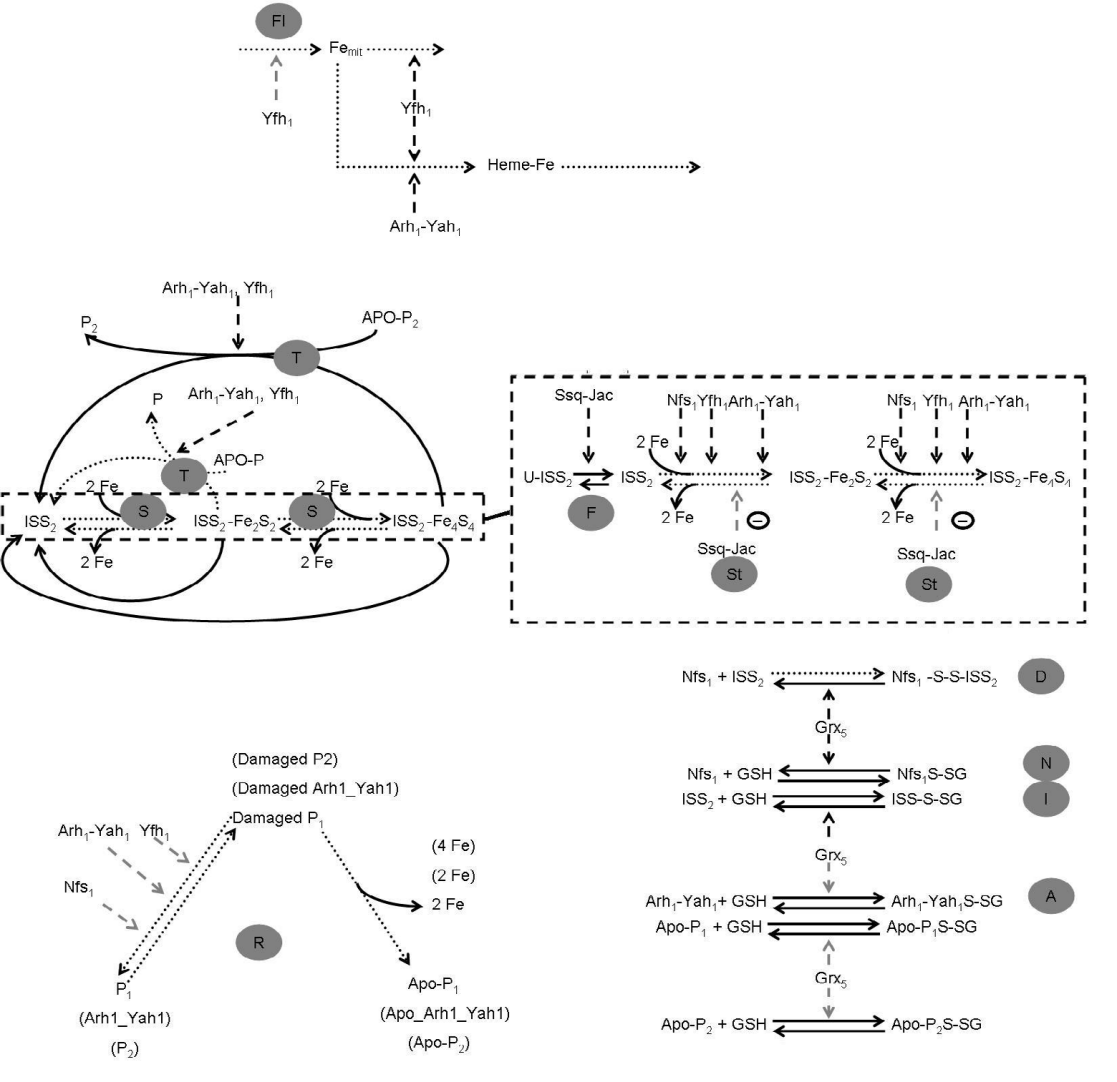
Network model for ISC biogenesis in *S. cerevisiae*. The letters in the grey circles stand for the different stage of the Biogenesis. **A **stands for the recovery of Arh1 by Grx5. **D **stands for the recovery of the dead end complex between Nfs1 and the scaffold by Grx5. **F **stands for folding. **FI **stands for Fe import. **I **stands for the recovery of the scaffolds by Grx5. **N **stands for Nfs1 recovery by Grx5. **R **stands for the repair of the clusters. **S **stands for the synthesis of ISC. **St **stands for stabilization of the ISC assembled on the scaffolds. **T **stands for the transfer of the ISC to apo-proteins. The species Apo-P and P in the transfer of a 2Fe2S cluster reaction represent the pairs Apo P1 and P1, Apo P2 and Fe2S2P2 or Fe2S2P2 and P2, respectively. See text and supplementary materials for details and for kinetic representation of the reactions. Dotted arrows represent reactions that have been observed to occur experimentally. Dashed arrows represent the alternative modes of regulatory action for the different proteins. Light grey arrows represent the network interactions that are not likely to exist in the pathway, according to our analysis.

The inherent complexity of the alternative reaction schemes in Figure 2 require the use of mathematical models in order to compare their *in silico *dynamic behavior against the phenotype reported in the literature for different genetically manipulated cell lines. However, the detailed mechanism of the processes and reactions in the various models are mostly unknown and appropriate parameter values are difficult to estimate. Therefore, to derive a useful mathematical model for each alternative network one needs to use approximate kinetic functions with parameters whose values can be bound to some extent. The power-law formalism, which is based on a Taylor approximation in a logarithmic space truncated in the first order term, provides a kinetic representation that a) is easily normalized and b) has parameter values boundaries that can be approximately estimated. This formalism and a scanning procedure similar to the one used here have previously been used to identify probable unknown regulatory interactions that were latter validated, in a detailed model of the red blood cell metabolism [70]. Furthermore, this formalism retains some non-linear character for the dynamics of the process under study [16-18,71].

We use the power-law formalism in its Generalized Mass (GMA) Action form for building-up a mathematical model that can accommodate the different alternatives in Figure 2. Details on the kinetic equations for our models, which are automatically generated from the network of reactions, are given in the supplementary appendix. The power-law description in GMA form of the network from Figure 2 is given in SBML format as supplementary material (see appendix for complete list of reactions and technical details of the approximation and parameter scanning procedure).

#### Normalization and parameter scanning

A power law model has two types of parameters. Multiplicative parameters are analogous to rate constants (γ_i_). They can be appropriately normalized and roughly determine the time scale of the processes. Apparent kinetic order *f*_*ij *_measures the direct influence (sensitivity) of metabolite *j *on flux *i*. Their values are typically smaller than the number of binding sites for the metabolite in the reaction, in absolute value [20,72]. Kinetic orders are positive if the flux is activated by the metabolite, negative if the flux is inhibited by the metabolite and zero if the considered metabolite has no direct influence upon the flux. The alternative networks we consider in order to evaluate the role of different proteins on ISC biogenesis can be derived as special cases of a general model that incorporates all the possible reactions and regulatory effects. Each alternative network is generated by setting to zero specific kinetic orders corresponding to proteins that do not directly modulate the flux of a given process in a specific network alternative. For example, when we consider that Grx5 may act upon recovery of Nfs1 activity by deglutathionylation, the kinetic order for Grx5 in the deglutathionylation flux is positive; otherwise this kinetic order is zero.

To analyze the behavior of each alternative network, we scan the relevant parameter values and evaluate the resulting dynamic behavior of the corresponding mathematical model. Because values for the kinetic orders have a limited range of permissible values, after normalization of the rate constants, one can do a fairly comprehensive scan of the parameter values for the alternative networks and characterize the most relevant behaviors associated with each topology.

**Figure 3:**
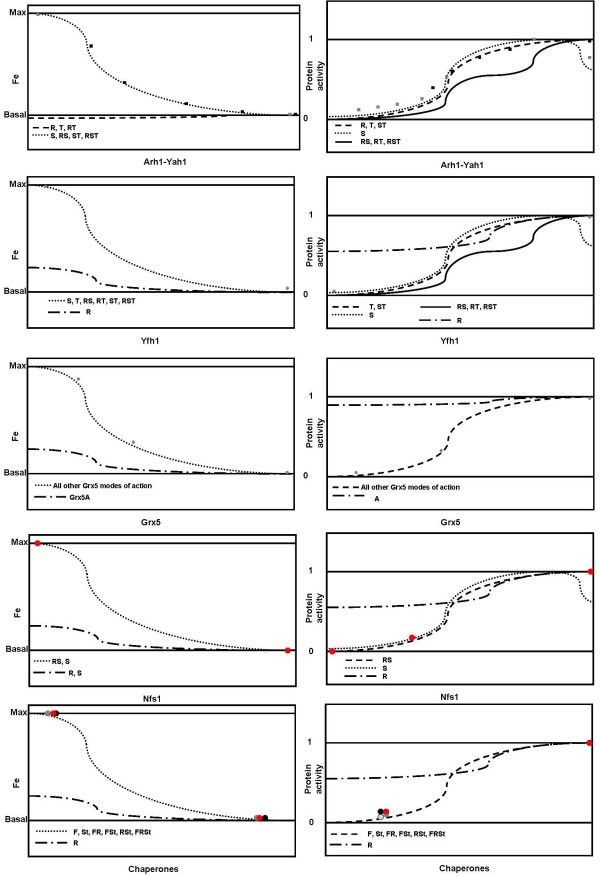
Comparison of the phenotypes in each of the alternative networks upon depletion of a given protein to the corresponding phenotype observed experimentally. Within each panel, we present the overall shapes of the curves that were obtained in our in silico simulations upon depletion of the target protein and points are presented to represent the curves of published experiments. Only in two cases were there more than two measurements made along the depletion curve. The curves are connected to the alternative networks according to the following labels. **R**: network where deleted protein acts only on repair. **F**: network where deleted protein acts only on folding. **S**: network where deleted protein acts only on synthesis. **St**: network where deleted protein acts only on stability of clusters. **A**: network where Grx5 acts on regulating glutathionylation state of Arh1. Any combination of labels indicates that the deleted protein in the network acts on more that one process. For example **RS **means that said protein acts on repair and synthesis of clusters. Panels A, C, D, E, G and I compare the accumulation of Fe in the network to that in the experiments. The Y-axis represents the level of free Fe. Panels B, D, F, H and J compare the evolution of ISC dependent protein activity in the alternative networks to that observed experimentally upon depletion of the protein. The Y-axis represents normalized ISC dependent protein activity. Panels A and B – Experimental data from [74] and [73]. Panels C and D – Experimental data from [88] and [87]. Panels E and F – Experimental data from [36]. Panels G and H – Experimental data from [125] and [126]. Panels I and J – Experimental data from [77, 127, 128].

Experimental semi-quantitative data about the effect of regulating the expression of the different genes involved in ISC assembly are available in the literature (see references in Figure 3). The following general methodology is used in the experiments reported in the literature:

1- The gene of interest is knocked out from the yeast genome and replaced with the same gene in a plasmid with expression controlled by either a repressible or an inducible promoter.

2- The phenotype of the mutated strain is compared to the wild type. This is done under conditions when the mutated strain synthesizes the protein of interest and repeated under conditions where the synthesis of that specific protein is shut off.

The experiments described in the previous paragraph can be reproduced in the following way, using our *in silico *models:

1- Introduce a sink flux for the protein of interest with the purpose of mimicking the shutting off of gene expression. For example, consider Grx5. When we are scanning to predict the role of Grx5, we define an auxiliary first-order reaction for Grx5 consumption. By changing the value of the rate-constant for this auxiliary reaction, we cause the amount of Grx5 to decrease as the simulation time increases. This reproduces the effect of the dilution and destruction of the protein in strains in which the gene has been knocked out [36].

2- Scan the parameter values and compute the resulting activity of ISC dependent enzymes, represented by P1 and P2 levels, and the mitochondrial iron levels. Rate constants are scanned for 3 orders of magnitude about each side of their normalized values. Details on the exact values for the scanning parameters are given in the supplementary appendix.

3- Save the simulation data. Plot it as is done in Figure 3. Verify if the concentration of Fe increases and the concentration of holo P1, holo P2, and holo Yah1 decreases upon the knock out of a specific gene, as observed experimentally.

As a result of this strategy, we obtain for each of the alternative networks a qualitative picture of the possible dynamic response regimes that the network may have. Following this methodology, the analysis of the *in silico *dynamic behavior of each network can have the following alternative outcomes: (i) A network reproduces the experimental depletion of ISC dependent protein activity and Fe accumulation that is observed in experiments upon knock out of a given protein, independent of parameter values; (ii) A network reproduces the experimental results for particular subsets of parameter values; (iii) A network does not reproduce the experimental results, independent of parameter values. Clearly, the last case means that the considered network is not a plausible representation of the target system. Designs that lead to reproducing observed results independently of the parameter choice are the most likely to be good approximations to the real network structure. Networks that reproduce experimental results only for particular parameter values are in between both extreme cases.

Using this approach, here we explore the role of the following proteins: (i) Arh1-Yah1, (ii) Yfh1, (iii) Grx5, (iv) Nfs1, and (v) Ssq1, Jac1 and Mge1. The results will help in discriminating between competing roles and in suggesting experiments to validate the predictions. Table 2 shows the possible roles each of the proteins in the scheme of Figure 2. All possible combinations of individual roles for each protein are also considered.

### The Role of Arh1-Yah1

The analysis from the bioinformatics section leads us to consider that Arh1-Yah1 can act in regulating **S**(ynthesis), **T**(ransfer) or **R**(epair) of the clusters (Table 2) or in any combination of the three roles. When comparing the simulations for the alternative roles of Arh1-Yah1 to the experimental results, we see that only the curves where Arh1-Yah1 participate in **S **or **ST **reproduce experimental results. Only for those roles does the activity of ISC dependent proteins decreases and Fe accumulates in response to a depletion in Arh1-Yah1 levels (Figure 3A and 3B). These results refine our previous predictions [44] and are in agreement with previous experimental results that have been interpreted as indicating that Arh1-Yah1 do not regulate ISC *in situ *repair [73,74].

It is interesting to note that the results from Arh1 depletion studies [74] show a slight increase in the activity of ISC-dependent proteins upon initial depletion of Arh1, followed by a decrease in ISC-dependent protein activity upon further depletion of Arh1. This behavior is not reproduced in Yah1 depletion studies [73]. Within the range of our parameter scanning, only the networks where Arh1-Yah1 acts on ISC synthesis shows the same behavior for the ISC-dependent proteins as that observed in Li *et al*. [74]. In our models this effect can be explained as follows. The simulations start with an overexpression of Arh1-Yah1. At high concentrations, much of the ISC biosynthesis takes place to keep Arh1-Yah1 active, as this protein requires ISC for activity. When the levels of Arh1-Yah1 drop bellow saturation levels the ISC biogenesis machinery can upload more ISC into the other ISC-dependent proteins, thus increasing their activity. As Arh1-Yah1 levels continue to drop there is not enough Arh1-Yah1 around to keep the full fraction of other ISC-dependent proteins in active form.

There is also the possibility that Arh1 and Yah1 act on ISC biogenesis in an unknown way that is not represented in the network. To look for additional evidence to support this hypothesis, we reanalyzed the phylogenetic profile of both proteins. This analysis reveals that Arh1 and Yah1 have a strong co-evolution with the proteins Sco1/Sco2 and Hol1, which are involved in cation transport. There are other connections between ISC biosynthesis and divalent ion mitochondrial transport, which suggests that the connection between Arh1/Yah1 and Sco1/Sco2 may be worth investigating in the future. Co-affinity purification experiments would provide evidence for this interaction. Additionally, measuring accumulation of iron and an impaired ISC dependent protein activity in mutants in which Sco1, Sco2 or Hol1 have been knocked out would provide evidence for the involvement of these proteins in ISC biogenesis.

We have also investigated the unsolved problem of the mutual functional relationship between Arh1 and Yah1. Homology with other ferredoxin/ferredoxin reductase pairs suggests that Arh1 is the reductase for Yah1. However, two-hybrid experiments have failed to recover an interaction between the two proteins [75], (Herrero, E. & Vilella, F. unpublished results). This negative result could be explained by the fact that the two-hybrid essay attempts to recover the interactions in a nuclear environment while the relevant interactions, if they exist, occur at the mitochondrial matrix.

Our structural modeling and protein docking experiments suggest that the two proteins interact in a way that is similar to their bovine homologues. We have repeated all the relevant analysis reported previously [44] for new structural models derived from updated structure information. We found that, although detailed numbers change, the general pictures remains the same. We have identified three arginine residues (Arg268, Arg272 and Arg273) in Arh1 that, upon mutation are likely to disturb Arh1-Yah1 interaction and thus reproduce both Δarh1 and Δyah1 mutant phenotypes [44]. This suggests a way of experimentally testing the predictions. If these proteins act together, disrupting this docking would affect their role in ISC biogenesis. This can be experimentally tested by creating point mutants of the two proteins, changing specific Arginine and Aspartate residues into alanine. Then, one could create mutant strains with different combinations of these two proteins. If the mutant strains accumulate Fe and show an impaired ISC dependent protein activity, this would be indirect evidence that the mutated residues are important for the function. Strains with tagged Arh1 and with tagged Yah1 could be used to over-express and purify the proteins by tag affinity purification (TAP). TAP analysis and co-purification experiments with the different point mutants of Arh1 and Yah1 should help in clarifying if Arh1 and Yah1 act in tandem during ISC biogenesis.

### The Role of Yfh1

The analysis from the bioinformatics section leads us to consider that Yfh1 can act in regulating Fe **I**(mport) and ISC **S**(ynthesis), **T**(ransfer) or **R**(epair) of the clusters (Table 2) or in any combination of the four roles. Only the curves for the simulations where Yfh1 participates in **S, T **or **ST **reproduce experimental results (Figure 3C and 3D). The activity of ISC dependent proteins decreases and Fe accumulates in response to the depletion of Yfh1, when Yfh1 acts in any of those roles. The phylogenetic and docking analysis supports that Yfh1 acts in S, T or ST because that analysis suggests that Yfh1 functionally cooperates with Arh1-Yah1. Recent experimental evidence detected physical and functional interactions between Yfh1, the scaffold proteins and Nfs1 [76-79] which provides further support for our interpretation. Other modes of action also cause accumulation of Fe and ISC dependent protein activity depletion; however either the Fe accumulation is small (**R**) or the protein activity depletion is biphasic (**RS, RT, RST**). Therefore we do not consider these as likely alternatives.

A speculative mechanistic model of how Arh1, Yah1 and Yfh1 may collaborate in ISC biogenesis is as follows. Yfh1 can regulate the oxidation state of iron ions, independently of the protein's oligomerization state [61,80,81]. Thus, Yfh1 could be responsible for supplying iron in the appropriate oxidation state for ISC biogenesis and repair. This has been previously suggested by Gakh *et al*. [80]. Arh1 and Yah1 can act as alternative electron donors/acceptors for Yfh1, ensuring Yfh1's ability to provide iron in the appropriate state of oxidation for synthesis and repair of ISC. The other donor/acceptor could be a mitochondrial respiratory chain. This alternative model could explain why some experiments detect independence of Yfh1 function from mitochondrial respiration [82] while others detect functional interaction between Yfh1 function and mitochondrial respiration [83]. One way to test this model would be the following. First, one would inhibit respiratory chain electron transfer (while still providing ATP for the cell) and Arh1-Yah1 independently, under the same experimental conditions. Yfh1 should remain active to some level in either case. Then, one would inhibit both simultaneously. If the model is correct then Yfh1 activity should be severely hindered in this double mutant cell lines.

Experimental evidence shows that, *in vitro*, Yfh1 can form large homo-multimers and store great amounts of iron that can be mobilized for later usage [81,84,85]. This is mimicked in our models by the role of Yfh1 in Section **I **of Figure 2. The analysis of these mathematical models shows that, if Yfh1 had such a function, Δyfh1 cells would have impaired ISC dependent protein activity. However, if the pool was as large as *in vitro *studies suggest it can be mitochondrial iron levels would be lower in Δyfh1 cells than in wild type cells. Thus, our simulations, together with recent experimental work [86], suggest that iron storage by Yfh1 is dispensable for its *in vivo *function.

### The Role of Grx5

The analysis from the bioinformatics section leads us to consider that Grx5 can act in regulating the **D**(ead end complex recycling), **A**(rh1-Yah1 deglutathionylation), **I**(SS) and **N**(fs1 deglutathionylation) blocks in Figure 2 (Table 2) or in any combination of the four roles. Only the simulation curves where Grx5 participates in regulating at least **D**, **I **or **N **are able to reproduce published experimental results (Figure 3E and 3F). Only the **A **mode of action for Grx5 can be excluded by our simulations.

*In silico *protein docking of Grx5 with each of the ISC proteins indicates that Nfs1 is the protein that has a better surface complementary with Grx5. Furthermore, the docking of Grx5 and Nfs1 suggests that Grx5 could be regulating the glutathionylation state of the Cys421 residue in Nfs1, which is the putative active site cysteine. This prediction could be tested by purifying and isolating the different proteins involved in ISC biogenesis, together with appropriate control proteins. Then one would incubate each protein with reduced glutathione, to create protein-GS adducts. In a different experiment, one would put the proteins under strong oxidizing conditions that lead to disulfide bridge formation. After that, one would mix each protein that has been submitted to oxidative stress with Grx5 and follow the reduction of the oxidized proteins. The targets for Grx5 should be the proteins that become reduced at a quicker rate. This experiment should clarify the role of Grx5 in ISC biogenesis. In parallel one could also perform co-affinity purification studies with Grx5 and identify which proteins interact with Grx5.

### The Role of Nfs1

Nfs1 catalyzes the removal of sulfur from cysteine and provides sulfur for ISC formation [29,87,88]. Nevertheless, it is not clear how it does so. It has been shown that Nfs1 transfers this sulfur to ISS proteins for ISC assembly [89,90]. It has also been shown *in vitro *that Nfs1 can directly repair ISC *in situ *upon oxidative damage, if iron is available in the ISC dependent protein [91-93]. However, it is unclear whether this later role is important in generating the phenotype of ISC deletion mutants. Thus we consider that Nfs1 can act in **S**(ynthesis) or **R**(epair) of the ISC (Figure 2, Table 2) or in both. The simulations show that the models can reproduce the experimental results if Nfs1 does not work on **R **(Figures 3G and 3H). Occam's razor suggests that participation of Nfs1 in **S **is sufficient to justify the phenotype. Nevertheless, the network where Nfs1 acts on **SR **can also reproduce the experimental phenotype observed upon Nfs1 depletion (Figure 3).

### The Role of the Chaperones and Co-chaperones Ssq1-Jac1-Mge1

We consider that Ssq1-Jac1-Mge1 can act in regulating **F**(olding of the proteins), **R**(epair of the clusters) and **St**(abilization of the clusters in the scaffolds) (Figure 2, Table 2) or in any combination of the three roles. When comparing the simulations for the alternative roles of the chaperones to published experimental results, only the curves where Ssq1-Jac1-Mge1 participate in regulating **R **can be excluded (Figure 3I and 3J). However, again following Occam's razor, there is no need to consider any role for the chaperones other than the folding of ISC proteins. This is in agreement with recently published work [94]. One way to test whether the chaperones are involved in the stabilization of the clusters in the scaffold proteins would be to create the following two *in vitro *systems. One system would contain scaffold proteins and Yah1. Then one would create favorable conditions so that the scaffolds would be loaded with ISC and follow the kinetics of both ISC assembly in the scaffolds and ISC transfer to apo-Yah1. This is an experiment that has been repeated and published using homologues from different species (see for example reference [95]). Finally, one would repeat the experiment adding chaperones to the medium. Faster kinetics of ISC assembly would suggest that the chaperones assist in stabilizing the clusters on the scaffolds.

## Discussion

The identification and reconstruction of novel pathways is an emerging area of great interest. The accumulation of data from different origins and the development of methods and software to mine that data create an opportunity to bridge the gap between the fragmentary view of genes and proteins and the more integrated approach of Systems Biology. In this paper we use a combination of theoretical, mathematical, and computational methods to reconstruct the topology of the reaction and regulatory network for the mitochondrial ISC biogenesis pathway in *S. cerevisiae*. The network elements (proteins) are identified and a network of interactions between them is predicted using automated literature mining, genomic data, structural bioinformatics data and evolutionary analysis. Although automatic tools do provide a first approximation of the network structure, human analysis remains necessary for curating all the relevant information. In fact, human curation of the network is a critical step for the derivation of possible alternative reaction schemes for the ISC biogenesis. At this point, mathematical models are needed to predict and validate the systemic behavior of each alternative network by comparing it to experimental data. Table 3 shows the likely roles for the proteins that have been analyzed. This eliminates some of the alternative network structures and assists in refining the remaining, by suggesting experiments that can further differentiate between them. These experiments are summarized in Table 4. As in any scientific approach, the process should continue iteratively.

As shown is this paper, bioinformatics tools, expert knowledge, and modeling techniques can thus be used to assist in the predictive analysis of the system, and in suggesting experimental test for the predictions. Another useful aspect of our approach is that it can be used in reassessing the interpretation of experimental data. For example, if Yfh1 had an important role in accumulating a mitochondrial Fe pool, then our model predicts that Δ*yfh1 *mutants would have less mitochondrial iron, which is opposite to what is observed. It is our goal to continue to refine our model and to apply this analysis to other organisms, thus assisting in the understanding of how the ISC biogenesis pathways have evolved.

**Figure 4:**
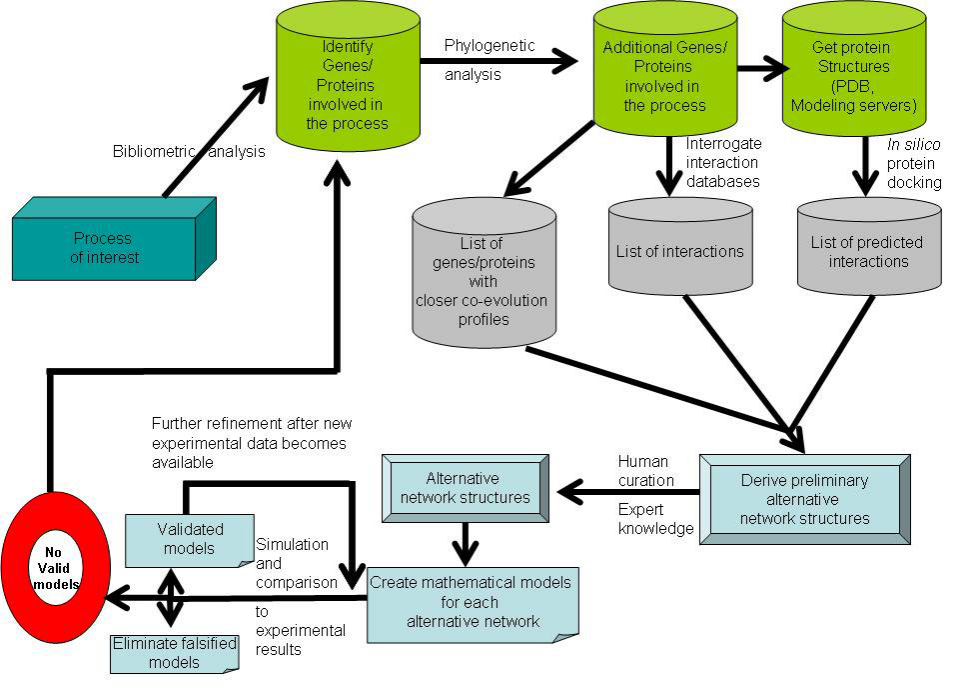
A simplified flow chart of the procedure suggested in the discussion. See discussion for details.

In principle, the combination of approaches presented here could be used in a flexible way to analyze similar problems in other molecular biological systems. In summary, the full procedure would be the following (Figure 4):

1) Choose the biological process of interest (in our benchmark example the proteins involved in ISC assembly in *Saccharomyces cerevisiae*)

2) Determine the proteins and metabolites of interest that are thought to be involved in the process (*Bibliomic analysis and Phylogenetic profiling*). Use phylogenetic profiling as a first indicator of which proteins may be acting together in the process of interest.

3) Interrogate available databases for physical interactions between the proteins of interest.

4) If possible, obtain structures for the proteins. If no structure is available, obtain structural models from homologue proteins (*Structural bioinformatics*)

5) Use all against all protein docking to derive the most likely interactions (*Structural bioinformatics*). Analyze those interactions (*Protein-protein data sets*).

6) Derive a degenerate/incomplete set of possible network structures based on the interactions obtained from 4).

7) Eliminate from/add to 5) any interactions that are eliminated/suggested from known data (*Expert knowledge*)

8) Identify alternative models corresponding to different hypothesis for the component elements and processes.

9) Derive mathematical models for the selected schemes using the GMA approach (*Mathematical modeling*)

10) Normalize the models and scan parameters over large permissible ranges to determine which alternative networks are able to reproduce known experimental behavior of the system.

11) If some of the alternatives reproduce known experimental results, devise thought experiments that can also be executed experimentally and that could differentiate between the behavior of the alternatives. Go back to step 6).

12) If none of the alternative networks is able to reproduce the known experimental behavior use Phylogenetic profiling to determine new potential components of the system of interest. Go back to step 3).

In certain cases, some of the steps proposed here can be avoided, as they would add no extra information. It is likely that, by applying this procedure to the reconstruction of different metabolic processes, the various methods will contribute differently for the reconstruction. In some cases, there will be overlap of the network of interactions that are predicted by each data set, while in others the analysis of the different datasets will provide different and sometimes contradictory networks of interactions. This emphasizes the need for expert curation of the networks at this stage of the methodology's development. Any program/server/software package that would allow the implementation of such a procedure would have to be sufficiently flexible so that the use of only certain parts of the procedure would be possible for non expert users. It is also critical that such an application is flexible enough to allow the researcher to modify the pathway topology interactively, using expert knowledge. Once the alternative pathways are identified, the use of GMA models greatly facilitates the mathematical modeling. These models can automatically be derived from the reaction scheme and dynamic simulations can be performed by scanning parameters of the normalized models. When it comes to ill-defined pathways with almost no parameter determination studies available, the scanning procedure is a limiting step, as tens of hundreds to a few millions of simulation curves may be needed even for fairly small pathways. The scanning of the parameter space can be bounded by the availability of good measurements for at least some of the parameter values. This is for example what happens in reference [70].

## Conclusion

The ISC biogenesis pathway of *S. cerevisiae *is partially reconstructed using a flexible *in silico *methodology that combines sequence analysis, literature analysis and structural bioinformatics methods. The role of different *S. cerevisiae *proteins in ISC biogenesis is predicted. Some predictions are validated by published experimental results. Other predictions need further experimental work to validate. The methodology proposed here is flexible and is applicable to the reconstruction of other systems. This methodology could be a step forward in integrating different types of data to a) obtain systemic knowledge about novel pathways, and b) clarify how current models of known pathways would work, generating rational hypothesis for testing.

## Methods

### Pathway Reconstruction from Automated Literature Analysis

Bibliometric methods consider that the co-occurrence of the names of two or more genes or proteins in the same paper is an indication that both proteins are likely to be involved in common cellular processes. Under this assumption one can identify proteins that are know to be involved in a given cellular process. ***iHOP ***[49] is a resource that allows this type of literature meta-analysis. We used this tool to identify possible additional genes involved in mitochondrial ISC biogenesis and as a first predictor of likely interactions between these different yeast proteins.

### Phylogenetic Profiling

This technique proposes that if the homologues of two proteins are equally present or absent in a set of fully sequenced genomes, then the two proteins are likely to be involved in some common cellular function [52,53,96]. The rationale for this assumption is that, given two proteins, they will co-evolve if some functional requirement of the organism is dependent on both in a similar way. To perform phylogenetic profiling of the proteins involved in mitochondrial ISC biogenesis we have downloaded the proteome of all fully sequenced organisms described in the KEGG database (Version 36.0). Homology searches for each of the *S. cerevisiae *proteins in each of the other proteomes was done by running version 2.2.4 of PSI-BLAST [97] locally, with e-value ≤ 0.0001 and three iterations. A vector of present or absent homologues in each genome was built for each yeast protein. Then, taking as reference the vector for the relevant protein, a co-occurrence index (CI) is calculated for each of the other proteins in the yeast genome: CI = Σ δ_ij-iPR_/Total number of genomes = 1-Normalized Hamming Distance between two genes. In this formula δ_ij-PR _is the Kronecker delta function, taken to be 1 if the reference protein PR and protein *j *both have (or do not have) homologues in the proteome of organism *i *and 0 otherwise. The Hamming distance has been used before and benchmarked both in *S. cerevisiae *and in other organisms [98]. We calculate the *p *value for each coefficient using the hypergeometric distribution as described in [99]. All CI values between any pair of ISC proteins have a p values smaller than10^-7^. Due to this, the *p*-value provides no discrimination for the likelihood of interaction between any pair of ISC biogenesis proteins. Thus, we selected as having a significant phylogenetic co-evolution any other ISC protein with a CI higher than that for 95% of all the proteins in the genome. Results are shown in Figure 1.

### Large Scale Protein Interaction Datasets

We have analyzed large scale protein interaction datasets downloaded from BIND [100], DIP [101-103], GRID [104,105], PathCalling [68,106] and YRC [107] to determine which proteins have been found to physically interact within the set of proteins involved in mitochondrial ISC biogenesis.

### Protein Modeling and Docking

Because the structure of the proteins involved in mitochondrial ISC biogenesis in *S. cerevisiae *has not been determined we had to create structural models to perform low resolution docking. These model have been obtained either by homology modeling, using 3DJIGSAW [108-114] and SWISSMODEL [115-118] or by *ab initio *modeling using ROSETTA [119-123]. Models were optimized locally by selected loop reconstruction using DEEPVIEW [115-118]. When more than one model was obtained for the same protein we used a genetic algorithm to optimize the structures [113], followed by loop reconstruction. Model optimization was finalized with a full energy minimization using the GROMACS97 force field [124] as implemented in DEEPVIEW. The protein models are given as supplementary material [See Additional File 2]

Given the atomic coordinates of two proteins, docking methods search for the best complex between them in which the shape of the two surfaces fit best [63,64], while also considering electrostatic interactions and hydrogen bonds. Protein docking experiments were done using GRAMM [54,55,57-60] and Hex [62]. The ability of these methods to recover existing complexes has been benchmarked by different authors [54,62] and, on a limited scale, by us for the ISC biogenesis pathway.

To calculate the docking score for a given pair of proteins, we used the list of the 20 highest docking scores from GRAMM. Within that list, we separated the clusters of docking solutions. The cluster score was obtained by adding the score of each individual solution within that cluster. Hex was then used to perform the same docking experiment. If the a solution from the cluster with the highest GRAMM score was not present in the best twenty Hex docking solutions, the GRAMM result was discarded and the score of the next best cluster that was also found by HEX was used instead. The results are shown in Figure 1. The scores are the product of the best fit solution within a cluster, predicted using GRAMM, by the size of the cluster within the 20 highest score predictions. In previous work, we performed several tests with positive and negative controls to ensure that this usage of the methods could indeed provide reasonable guesses. For negative controls of the docking we chose Grx5. We obtained structures for the proteins Rex2, Cyt1, Lip2, Atp16 and Cox 11. These are all proteins that either are not located in the mitochondria or, if located in the mitochondria, are not involved in ISC biogenesis. Then, we performed docking of Grx5 against these proteins as well as against the ISC biogenesis proteins. The results rank the five negative controls as the 5 smallest docking scores [34]. As positive control we performed pair wise docking of Arh1, Yah1 and their bovine homologues. The test scores are higher for the appropriate pairs (Arh1-Yah1 and Bovine Ferredoxin Reductase-Bovine Ferredoxin; data not shown). Furthermore, the best docking for the bovine pair had a RMSD of less than three Angstroms with respect to the crystallized structure of the complex. This suggests that the docking methods used here are useful for the purpose we describe.

### Mathematical Modeling

In this paper the generalized mass action (GMA) canonical representation of the power-law formalism is used for the set of ordinary differential equations that describes the dynamical behavior of the metabolic network of interest. The derivation and normalization of the models using this formalism is explained in detail in the supplementary appendix. The complete model is provided as supplementary material [see Additional File 3]. The GMA models are used to predict the dynamic behavior of the alternative networks. This behavior is then compared to that observed in published experiments and network alternatives are validated or invalidated on the basis of being able or unable (respectively) to reproduce published those experimental results.

The experimental data regarding changes in concentration for this system are mostly qualitative or semi quantitative and incomplete. For example when there are measurements at several time points for the accumulation of iron or for the decrease of ISC dependent protein activity, there are no measurements regarding the decrease in concentration of the protein being knocked out of the genome. Also, in most cases, there are only two measurements for Fe concentrations and ISC dependent protein activity. One measurement is made in wild type cells and the other measurement in the mutated strains at a suitable time point. This precludes using any current fitting algorithm with precision to determine goodness of fit between the dynamic behavior of a given topology and the experimental results. If accurate quantitative data regarding the concentrations of the different proteins, the variation of Fe concentration and ISC dependent protein activity were available, we would apply such a fitting algorithm to find the set(s) of parameter values that would best fit those data. For each alternative topology, the least square deviation between the observed data and the best fit set(s) of parameter values could then be used to determine which topology would be more likely to explain the experimental data. Ranking the more likely models in the absence of accurate experimental data is less straight forward. If no topology can reproduce experimental results over the entire range of scanned parameter values one can calculate the percentage of the parameter space in which the experimental behavior is qualitatively reproduced. The higher that percentage, the more likely it is that a given topology is able to explain the experimental results. However if more than one topology reproduces the qualitative experimental observations over the entire range of parameter values that are scanned, it is difficult to define a measure for the goodness of fit of these topologies to the experimental data.

## Authors' contributions

RA designed the study, performed the computational work, analyzed the data and wrote the paper. AS analyzed the data and wrote the paper. Both authors read and approved the final manuscript.

## Supplementary Material

Additional File 1 Supplementary Appendix. Appendix detailing the curation of the reaction schemas and the creation of the power law models. Click here for file

Additional File 2 Structural models used for the docking studies of the ISC biogenesis proteins. Structural models used for the docking studies of the ISC biogenesis proteins in PDB format. Click here for file

Additional File 3 Model for the ISC Biogenesis. The SBML representation of the model networks that were used to generate Figure 3. Click here for file

## Figures and Tables

**Table 1:** Proteins involved in ISC synthesis in *Saccharomyces cerevisiae*.

Proteins	Protein Function	Type of evidence for Protein Function	Suggested Function In ISC Assembly	Type of evidence for suggested systemic role
Arh1	Ferredoxin Reductase	Homology to known ferredoxin/adrenodoxin reductases	Reduces Yah1/Provides electrons for ISC assembly/transfer/repair	Homology, Physiological, Genetic
Yah1	Ferredoxin	Homology to known ferredoxin/adrenodoxin	Provides electrons for ISC assembly/transfer/repair	Physiological, Genetic
Yfh1	Frataxin	Homology, Biochemical; Structural	Stores/Provides Fe directly to ISC assembly and Heme synthesis	Physiological, Structural, Genetic
Grx5	Glutaredoxin	Homology, Biochemical	Regulates glutathionylation state of protein cysteinyl residues	Physiological, Biochemical
Isa1	Scaffold		As dimer scaffolds initial ISC assembly between two monomers and then transfers it to apo-proteins	Biochemical, Spectroscopic, Direct transfer observation
Isa2	Scaffold		As dimer scaffolds initial ISC assembly between two monomers and then transfers it to apo-proteins	Biochemical, Spectroscopic, Direct transfer observation
Isu1	Scaffold		As dimer scaffolds initial ISC assembly between two monomers and then transfers it to apo-proteins	Biochemical, Spectroscopic, Direct transfer observation
Isu2	Scaffold		As dimer scaffolds initial ISC assembly between two monomers and then transfers it to apo-proteins	Biochemical, Spectroscopic, Direct transfer observation
Nfu1	Scaffold		As dimer scaffolds initial ISC assembly between two monomers and then transfers it to apo-proteins	Biochemical, Spectroscopic
Ssq1	Hsp70 Chaperone	Homology, Biochemical, Genetic, Structural	Assists in proper folding of ISC biosynthetic proteins, namely Yfh1 and Isa-Isu proteins/Assists in maintaining ISC assembled in scaffold dimer for proper transfer	Biochemical, Structural
Jac1	Hsp40 Co- chaperone	Homology, Biochemical, Genetic, Structural	Assists Ssq1 in interacting with Isu/Isa proteins	Biochemical, Genetic, Structural
Mge1	Co- chaperone/Nucleotide exchange factor	Homology, Biochemical, Genetic, Structural	Assists Ssq1	Biochemical, Genetic, Structural
Nfs1	Cysteine Desulfurase	Homology, Biochemical	Provides sulphur for ISC assembly in scaffold dimers or *in situ *ISC assembly/repair	Physiological, Biochemical, Genetics
Atm1	ABC transporter	Homology	Involved in ISC export for cytoplasm and nuclear proteins	Physiological, Genetic
Isd11	unknown	Biochemical, Genetic	Fundamental for Nfs1 action	Physiological, Genetic

**Table 2:** Possible alternative regulatory roles for the different proteins in the scheme shown in Figure 2. A '+' sign in a row indicates that the protein could participate in the regulation of reactions in the corresponding block of reactions in Figure 2.

Sites in Figure 2	Proteins				
	Arh1-Yah1	Yfh1	Nfs1	Ssq1-Jac1	Grx5
R	**+**	**+**	**+**		
S	**+**	**+**	**+**		
T	**+**	**+**			
FI		**+**			
St				**+**	
F				**+**	
D					**+**
I					**+**
N					**+**
A					**+**

**Table 3:** Roles for the proteins in ISC biogenesis. Likely roles for the different proteins in the scheme shown in Figure 2 are indicated by a '+' sign. The letter codes refer to the blocks in Figure 2. Entries not in bold are not likely due to Occam's razor rule, although they reproduce the experimental results.

	Proteins				
	Arh1-Yah1	Yfh1	Nfs1	Ssq1-Jac1	Grx5

**Roles**	**S**	**S**	**S**	**F**	**D**
	**ST**	**T**	**SR**	**St**	**I**
		**ST**		**FSt**	**N**
				FR	**DI**
				RSt	
				FRSt	**DN**
					**IN**
					**DIN**
					ADIN

**Table 4:** Experimental validation of the predictions and suggested experiments. See text for details on the suggested experiments.

**Prediction**	**Experimental Validation**	**Suggested experiments**
Grx5 acts in recovering the activity of Nfs1 or of the scaffold proteins	Experimentally detected interaction with the scaffolds [34]	Use of kinetic assays and TAP assays to pinpoint the most likely targets for Grx5
Arh1-Yah1 act on **S **or **ST**	Yes [73, 74]	
Arh1-Yah1 interact in a similar way to their bovine homologues	No	Point Mutants and use of TAP assays combined with measurements of ISC dependent protein activity and Fe accumulation in mutant strains
Yfh1 acts on **S**, **T **or **ST**	Yes [76-79]	
Yfh1 storage of Fe is not important for its ISC biogenesis role	Yes [86]	
Nfs1 role in synthesis is sufficient to justify Δnfs1 phenotype	No	
Chaperone role in **F **or **St**	Yes for **F **[94]	*In vitro *systems with and without chaperones to compare rate of assembly of ISC onto the scaffold would provide some evidence for or against **St**.
