# Supplementary material for: In silico pathway reconstruction: Iron-sulfur cluster biogenesis in Saccharomyces cerevisiae
Source: BMC Syst Biol. 2007 Jan 31;1:10. doi: 10.1186/1752-0509-1-10 (PMC1839888; doi:10.1186/1752-0509-1-10)
Supplement: Additional File 1 — Supplementary Appendix. Appendix detailing the curation of the reaction schemas and the creation of the power law models. [file 1752-0509-1-10-S1.pdf]

Supplementary Appendix

Generating a curated pathway model using expert knowledge

After an analysis of the relevant literature, the following detailed information was considered to build the networks shown in Figure 2. Bold letters shown parenthetically identify which part of Figure 2 is described:

i) Iron is imported into the mitochondrial matrix. Once at the mitochondrial matrix, iron is loaded directly into the enzyme ferrochelatase [1, 2] and is directed for heme synthesis and ISC biogenesis. Some results show that, *in vitro*, Fe can also be stored by frataxin (Yfh1) for posterior usage [3, 4]. The *in vivo* relevance of this is disputed. To a first approximation one can simplify the reaction network by assuming that a mitochondrial iron pool exists and that the iron from this pool is used for both heme A synthesis and for ISC synthesis. We also consider a production flux that accounts for iron import into that mitochondrial pool and a sink flux that accounts for any other usage or export of iron from the mitochondria. According to the available information, possible alternative roles for Yfh1 in Fe processing are: a) Regulation of Fe import and usage (**I**) and b) Regulation of ISC synthesis (**S**), transfer (**T**) and repair (**R**) through the regulation of Fe supply [5, 6].

ii) ISC initially assemble in the Isu1, Isu2, Isa1, Isa2, and Nfu1 scaffold dimers, represented by ISS in Figure 2 (e. g. [7]). Scaffolds form homodimers where the initial ISC assembly takes place [6, 8]. ISC synthesis (**S**) is catalyzed by cysteine desulfurase Nfs1 [9-11] and requires electron balancing. One possible role for Arh1-Yah1 in the ISC synthesis (**S**) process is that of electron transfer regulation. Recent evidence suggests that, in *E. coli*, the

Isa1/Isa2 homologue protein IscA is important in providing Fe to the Isu1/Isu2 homologue protein [12-15]. In our model, for simplicity's sake, we will not differentiate between different types of scaffold proteins.

iii) Once a 2Fe-2S ISC is assembled in the scaffold it can be transferred (**T**) to 2Fe-2S apo-proteins[12, 13, 16-21], represented in Figure 2 by Apo-P1 and Apo_Arh1_Yah1. There is also the possibility that 2Fe-2S clusters are transferred (**T**) to 4Fe-4S apo-proteins [16, 18, 19]. Two transfer steps would then lead to the formation of the appropriate 4Fe-4S ISC on the apo-protein. The affinity of the clusters for the scaffolds (and thus the transfer of the ISC) is modulated by the reduction state of the cluster [22]. Herein lays another possible role for Arh1-Yah1 in ISC biogenesis. These proteins could provide electrons to regulate the process of ISC transfer (**T**) to Apo-proteins.

iv) If the ISC remains on the scaffold proteins, it can be transformed into a 4Fe-4S ISC [8, 23]. Roles for Yfh1, Nfs1, and Arh1-Yah1 in this additional ISC synthesis (**S**) step are similar to those described before.

v) Although we know of no direct evidence for this, one can not rule out the possibility that the 4Fe-4S ISC can be transferred directly to 4Fe-4S apo-proteins, represented in Figure 2 by Apo P2. Arh1-Yah1 could have a role in providing electrons to regulate cluster affinity and transfer (**T**).

vi) There is a natural turnover of ISC, both in scaffold proteins and in the ISC proteins, for example due to oxidative stress [e. g. [24, 25]]. Nfs1 homologues are able to repair (**R**) damaged ISC directly *in situ* [23, 26]. A role for Arh1-Yah1 in providing electrons to facilitate this repair (**R**) is possible.

vii) Finally, once assembled in the scaffold proteins, the ISC can be transferred to the cytoplasm [27, 28], most likely through Atm1.

viii) Grx5 is a monothyolic reductase that catalyzes the reaction $P - SSG + GSH \leftrightarrow PSH + GSSG$ [29]. Because ISC are coordinated between cysteine residues, glutathionylation of such residues would prevent formation of ISC and thus disturb normal ISC biogenesis and ISC dependent protein activity. Thus, Grx5 could be active in regulating the glutathionylation state of cysteine residues in Arh1, Yah1, scaffold or Nfs1 proteins. Grx5 could also be involved in regulating the formation/destruction of disulfide bridges in these proteins [29]. These two modes of action are lumped into one by defining for each ISC assembly protein an inactivated pool that can be reactivated by Grx5.

ix) There is a possibility that Grx5 protein-protein disulfide bridge reducing activity could act upon such bridges formed between different ISC proteins. There have been reports that, in the absence of iron on the scaffold dimers, such a bridge forms between Isu and Nfs1 homologues, leading to a dead end complex between the two proteins [30-32]. Grx5 could be active in reducing these bridges and returning both proteins for active duty in ISC assembly.

x) The HSP70-type protein chaperone Ssq1 is important for proper folding of the proteins involved in ISC biogenesis pathway and for the proper functioning of the pathway. Mge1 is used by Ssq1 as a nucleotides exchanging factor. Jac1 is a HSP40-type co-chaperone homologue that is important for the appropriate functioning of Ssq1 in the ISC biogenesis pathway. Ssq1, Mge1 and Jac1 work together and are activated by Isu

homologues [7, 33-39], which suggests that these proteins may be involved in: a) stabilizing (**St**) ISC assembly in the scaffolds until a productive transfer occurs [7, 40], b) in initial folding (**F**) of scaffold proteins or other ISC proteins or c) in both processes.

Derivation of the GMA Model

a) The power-law approximation:

If the flux of a given process is regulated by species X_1, X_2, …, X_n but the functional form of the rate expression is unknown one can write the following equation to describe its rate.

$$v_j = F_j\left(X_1, X_2, ..., X_n\right) \qquad \text{Eq. 1}$$

In general, the exact form of *F* is unknown. If one assumes that this function is a rational function [7, 40-42], one can write the same equation in logarithmic space and approximate that function using a Taylor series. Then, by truncating the series in the first order term and returning to a Cartesian space, the unknown form of the function can be approximated by a power-law function [43, 44]:

$$v_j \cong \gamma_j \prod_{k=1}^{i} X_k^{f_{j,k}} \qquad \text{Eq. 2}$$

Where γ_j is an apparent rate constant and $f_{j,k}$ an apparent kinetic order:

$$\gamma_j = v_{j,0} \prod_{k=1}^{i} X_{k0}^{f_{j,k}}$$

$$f_{j,k} = \left.\frac{dLog\left[v_j\right]}{dLog\left[X_k\right]}\right|_0$$

In the case of the ISC biogenesis, the reproduction of experimental results requires that the concentration of several dependent variables decreases steadily until it ultimately is zero, for example when reproducing a Grx5 knock-out cell line. Many of the rate expressions approach zero as the concentrations of different species decrease, which suggest that the Generalized Mass Action (GMA) representation is more adequate for our models. By using such a representation, we ensure that as one protein is knocked out of the model it will affect only the process it is supposedly involved in.

b) Reactions that constitute a base model for ISC biogenesis

The reactions used in the model, together with rate expressions are shown in Supplementary Table 1. The assemble model and differential equations are given as supplementary material in an SBML file. This model is obtained in the following way. First, the mass balance equations for each internal variable are written. For instance, in the case of the pool of scaffold proteins (ISS in Figure 2), we have the following processes:

Biogenesis of the Fe2S2 cluster	2 Fe + ISS → ISSFe2S2	$v_3 = \gamma_3 Fe^{f31} ISS^{f32} [Arh1\_Yah1^{f33}][Nfs1^{f34}][Yfh1^{f35}]$
Loss of the Fe2S2 cluster	ISSFe2S2 → 2 Fe + ISS	$v_4 = \gamma_4 ISSFe_2S_2^{f41} [SsqJac^{f42}]$
Unfolding of the scaffolds	U_ISS → ISS	$v_7 = \gamma_7 ISS^{f71} [SsqJac^{f72}]$
Folding of the scaffolds	ISS → U_ISS	$v_8 = \alpha_8 ISS$
Transfer of the Fe2S2 cluster	ISSFe2S2 + Apo_P1 → ISS + P1	$v_9 = \gamma_9 Apo\_P1^{f91} ISSFe_2S_2^{f92} [Arh1\_Yah1^{f93}][Yfh1^{f94}]$
Transfer of the Fe4S4 cluster	ISSFe4S4 + Apo_P2 → ISS + P2	$v_{10} = \gamma_{10} Apo\_P2^{f101} ISSFe_4S_4^{f102} [Arh1\_Yah1^{f103}][Yfh1^{f104}]$
Transfer of the Fe2S2 cluster	ISSFe2S2 + Apo_P2 → ISS + Apo1_P2	$v_{11} = \gamma_{11} Apo\_P2^{f111} ISSFe_2S_2^{f112} [Arh1\_Yah1^{f113}][Yfh1^{f114}]$
Transfer of the Fe2S2 cluster	ISSFe2S2 + Apo1_P2 → ISS + P2	$v_{12} = \gamma_{12} Apo1\_P2^{f121} ISSFe_2S_2^{f122} [Arh1\_Yah1^{f123}][Yfh1^{f124}]$
Transfer of the Fe2S2 cluster	ISSFe2S2 + Apo_Arh1_Yah1 → ISS + Arh1_Yah1	$v_{13} = \gamma_{13} Apo\_Arh1\_Yah1^{f131} ISSFe_2S_2^{f132} [Arh1\_Yah1^{f133}][Yfh1^{f134}]$
Glutathionylation of Scaffold	ISS → ISS_SG	$v_{22} = \gamma_{22} ISS^{f221}$
Deglutathionylation of Scaffold	ISS_SG → ISS	$v_{23} = \gamma_{23} ISS\_SG^{f231} [Grx5^{f232}]$
Loss of Fe4S4 cluster from scaffold	ISSFe4S4 → ISS + 4 Fe	$v_{35} = \gamma_{35} ISSFe_4S_4$

Thus, the mass action term for ISS is:

$$\frac{dISS}{dt} = v_4 - v_3 + v_7 - v_8 + v_9 + v_{10} + v_{11} + v_{12} + v_{13} - v_{22} + v_{23} + v_{35}$$

Then, each velocity is substituted by its power-law form according to the information provided in the Supplementary Table 1. Protein and metabolite levels are considered at their basal steady-state. Then, the apparent rate constants are also normalized. This allows for a more appropriate comparison of the different results.

c) Scanning procedure

The scanning procedure considers different values for the parameters as indicated in Supplementary Table 2. A value of 0 in the corresponding kinetic-order removes the considered variable from the model. Results are computed using Mathematica©.
The values for the scanned parameters are given in Supplementary Table 2. Due to the combinatorial explosion of possibilities some simplifying assumptions were made for the scanning. Rate constants for similar processes are scanned as being the same. The same applies to the kinetic orders. This is all indicated in Supplementary Table 2. Another simplifying assumption that we made was that while scanning for the role of one protein, the kinetic orders for the remaining proteins remained constant. Thus, for example when studying the role of Arh1-Yah1, the kinetic orders that regard the role of Grx5 were left untouched and with a value of 1. The rate constant for the sink reaction of Arh1 was set to 0.1 while the rate constants for all other sink reactions were zero. The values for the rate constants of reactions in which Arh1-Yah1 was not involved were left at their basal values which are reported in the SBML file supplied as

supplementary material. We performed in excess of two and half million simulations curves for the scanning.

Supplementary Table 1: Set of Reactions in the complete model

Reaction	Description	Chemical Equation [a]	Rate Expression [b]
1	Iron entry into the mitochondria	→ Fe	$v_1 = \gamma_1 [Yfh1^{f_{11}}]$
2	Iron sink for other processes and export	Fe →	$v_2 = \gamma_2 Fe^{f21}$
3	Biogenesis of the Fe2S2 cluster	2 Fe + ISS → ISSFe2S2	$v_3 = \gamma_3 Fe^{f31} ISS^{f32} [Arh1\_Yah1^{f33}][Nfs1^{f34}][Yfh1^{f35}]$
4	Loss of the Fe2S2 cluster	ISSFe2S2 → 2 Fe + ISS	$v_4 = \gamma_4 ISSFe_2S_2^{f41} [SsqJac^{f42}]$
5	Biogenesis of the Fe4S4 cluster	ISSFe2S2 + 2 Fe → ISSFe4S4	$v_5 = \gamma_5 Fe^{f51} ISSFe_2S_2^{f52} [Arh1\_Yah1^{f53}][Nfs1^{f54}][Yfh1^{f55}]$
6	Loss of the Fe4S4 cluster	ISSFe4S4 → ISSFe2S2 + 2 Fe	$v_6 = \gamma_6 ISSFe_4S_4^{f61} [SsqJac^{f62}]$
7	Unfolding of the scaffolds	U_ISS → ISS	$v_7 = \gamma_7 ISS^{f71} [SsqJac^{f72}]$
8	Folding of the scaffolds	ISS → U_ISS	$v_8 = \alpha_8 ISS$
9	Transfer of the Fe2S2 cluster	ISSFe2S2 + Apo_P1 → ISS + P1	$v_9 = \gamma_9 Apo\_P1^{f91} ISSFe_2S_2^{f92} [Arh1\_Yah1^{f93}][Yfh1^{f94}]$
10	Transfer of the Fe4S4 cluster	ISSFe4S4 + Apo_P2 → ISS + P2	$v_{10} = \gamma_{10} Apo\_P2^{f101} ISSFe_4S_4^{f102} [Arh1\_Yah1^{f103}][Yfh1^{f104}]$
11	Transfer of the Fe2S2 cluster	ISSFe2S2 + Apo_P2 → ISS + Apo1_P2	$v_{11} = \gamma_{11} Apo\_P2^{f111} ISSFe_2S_2^{f112} [Arh1\_Yah1^{f113}][Yfh1^{f114}]$
12	Transfer of the Fe2S2 cluster	ISSFe2S2 + Apo1_P2 → ISS + P2	$v_{12} = \gamma_{12} Apo1\_P2^{f121} ISSFe_2S_2^{f122} [Arh1\_Yah1^{f123}][Yfh1^{f124}]$
13	Transfer of the Fe2S2 cluster	ISSFe2S2 + Apo_Arh1_Yah1 → ISS + Arh1_Yah1	$v_{13} = \gamma_{13} Apo\_Arh1-Yah1^{f131} ISSFe_2S_2^{f132} [Arh1\_Yah1^{f133}][Yfh1^{f134}]$
14	Partial degradation of the Fe2S2 cluster	Arh1_Yah1 → D_Arh1_Yah1	$v_{14} = \gamma_{14} Arh1\_Yah1$
15	Repair of the Fe2S2 cluster	D_Arh1_Yah1 → Arh1_Yah1	$v_{15} = \gamma_{15} D\_Arh1\_Yah1^{f151} [Nfs1^{f152}][Arh1\_Yah1^{f153}][Yfh1^{f154}][SsqJac^{f155}]$
16	Partial degradation of the Fe2S2 cluster	P1 → D_P1	$v_{16} = \gamma_{16} P1$
17	Repair of the Fe2S2 cluster	D_P1 → P1	$v_{17} = \gamma_{17} D\_P1^{f171} [Nfs1^{f172}][Arh1\_Yah1^{f173}][Yfh1^{f174}][SsqJac^{f175}]$
18	Loss of inactive Fe2S2 cluster	Apo1_P2 → Apo_P2 + 2Fe	$v_{18} = \gamma_{18} Apo1\_P2$

19	Loss of inactive Fe2S2 cluster	D_Arh1_Yah1 → Apo_Arh1_Yah1 + 2 Fe	$v_{19} = \gamma_{19} D\_Arh1\_Yah1$
20	Loss of inactive Fe2S2 cluster	D_P1 → Apo_P1 + 2 Fe	$v_{20} = \gamma_{20} D\_P1$
21	Loss of inactive Fe4S4 cluster	D_P2 → Apo_P2 + 4 Fe	$v_{21} = \gamma_{21} D\_P2$
22	Glutathionylation of Scaffold	ISS → ISS_SG	$v_{22} = \gamma_{22} ISS^{f221}$
23	Deglutathionylation of Scaffold	ISS_SG → ISS	$v_{23} = \gamma_{23} ISS\_SG^{f231} [Grx5^{f232}]$
24	Glutathionylation of P1	Apo_P1 → P1_SG	$v_{24} = \gamma_{24} Apo\_P1^{f241}$
25	Deglutathionylation of P1	P1_SG → Apo_P1	$v_{25} = \gamma_{25} P1\_SG^{f251} [Grx5^{f252}]$
26	Glutathionylation of P2	Apo_P2 → P2_SG	$v_{26} = \gamma_{26} Apo\_P2^{f261}$
27	Deglutathionylation of P2	P2_SG → Apo_P2	$v_{27} = \gamma_{27} P2\_SG^{f271} [Grx5^{f272}]$
28	Glutathionylation of Arh1_Yah1	Apo_Arh1_Yah1 → Arh1_Yah1_SG	$v_{28} = \gamma_{28} Apo\_Arh1\_Yah1^{f281}$
29	Deglutathionylation of Arh1_Yah1	Arh1_Yah1_SG → Apo_Arh1_Yah1	$v_{29} = \gamma_{29} Arh1\_Yah1\_SG^{f291} [Grx5^{f292}]$
30	Formation of dead-end complex	Nfs1 + ISS → Nfs1_ISS	$v_{30} = \gamma_{30} Nfs1\, ISS$
31	Recovery of the dead-end complex	Nfs1_ISS → Nfs1 + ISS	$v_{31} = \gamma_{31} Nfs1\_ISS^{f311} [Grx5^{f312}]$
32	Glutathionylation of Nfs1	Nfs1 → Nfs1_SG	$v_{32} = \gamma_{32} Nfs1$
33	Deglutathionylation of Nfs11	Nfs1_SG → Nfs1	$v_{33} = \gamma_{33} Nfs1\_SG^{f331} [Grx5^{f332}]$
34	Loss of Fe4S4 cluster from scaffold	ISSFe4S4 → ISS + 4 Fe	$v_{35} = \gamma_{35} ISSFe_4S_4$
35	Partial degradation of the Fe4S4 cluster	P2 → D_P2	$v_{35} = \gamma_{35} P2$
36	Repair of the Fe4S4 cluster	D_P2 → P2	$v_{36} = \gamma_{36} D\_P2^{f361} [Nfs_1^{f362}][Arh1\_Yah1^{f363}][Yfh1^{f364}][SsqJac^{f365}]$
37	Export of Fe2S2 cluster to the cytoplasm	ISSFe2S2 →	$v_{37} = \gamma_{37} ISSFe_2S_2^{f371}$
38	Export of Fe4S4 cluster to the cytoplasm	ISSFe4S4 →	$v_{38} = \gamma_{38} ISSFe_4S_4^{f381}$
39	Formation of Heme	Fe → Heme_Fe	$v_{39} = \gamma_{39} Fe^{f391} [Arh1\_Yah1^{f392}][Yfh1^{f393}]$
40	Destruction of Heme	Heme_Fe → Fe	$v_{40} = \gamma_{40} Heme\_Fe^{f401}$

41	Depletion of Yfh1 for Δyfh1 cells	$Yfh_1 \rightarrow$	$v_{41} = \gamma_{41} Yfh1$
42	Depletion of Arh1_Yah1 for Δarh1 and Δyah1 cells	Apo_Arh1_Yah1→	$v_{42} = \gamma_{42} Arh1\_Yah1$
43	Depletion of scaffold for Δssq1 and Δjac1 cells	SsqJac→	$v_{43} = \gamma_{43} SsqJac$
44	Depletion of Nfs1 for Δnfs1 cells	Nfs1→	$v_{44} = \gamma_{44} Nfs1$
45	Depletion of Grx5 for Δgrx5 cells	Grx5→	$v_{45} = \gamma_{45} Grx5$

a ISS – Scaffold for initial ISC assembly; ISSFe2S2, ISSFe4S4 – Scaffold with a Fe2S2 and a Fe4S4 ISC cluster assembled, respectively; Fe – Mitochondrial iron; P1 – generic protein that needs a Fe2S2 ISC to be functional; P2 – generic protein that needs a Fe4S4 ISC to be functional; Arh1_Yah1 – electron donor (either Arh1 or Yah1; see text for an explanation); Apo_P1, Apo_P2, Apo_Arh1_Yah1 –apo forms of P1, P2 and Arh1_Yah1 respectively; P1_SG, P2_SG, Arh1_Yah1_SG, Nfs1_SG, Isu_SG – glutathionylated forms of P1, P2, Arh1_Yah1, Nfs1 and Isu, respectively; D_P1, D_P2, D_Arh1_Yah1, - Forms of P1, P2 and Arh1_Yah1, respectively, with a damaged and repairable ISC; Apo1_P2 – P2 form with an intermediate Fe2S2 cluster assembled; Heme – heme molecules synthesized in the mitochondrial matrix; Heme_Fe – Heme molecules with iron.

b Species in parenthesis and brackets in the equations are modifiers and are not represented in the flux diagram because they contribute to the catalysis of the reaction but are neither produced nor consumed in the reaction

Supplementary Table 2: Parameters that were scanned and ranges of values for the scanning. A total of 5647152 simulations were done.

Parameters	Type of parameter	Scanning Range	Controlled processes
$\gamma_3 = \gamma_5$	Rate constant	$10^{-3} - 10^{2}$ 7 samples uniformly distributed in Log space	Assembly of ISC in scaffold
$\gamma_4 = \gamma_6 = \gamma_{35}$	Rate constant	$10^{-3} - 10^{2}$ 7 samples uniformly distributed in Log space	Loss of ISC from scaffold
γ_8	Rate constant	$10^{-3} - 10^{2}$ 7 samples uniformly distributed in Log space	Rate of appropriate folding for scaffolds
$\gamma_9 = \gamma_{10} = \gamma_{11} = \gamma_{12} = \gamma_{13}$	Rate constant	$10^{-3} - 10^{2}$ 7 samples uniformly distributed in Log space	ISC transfer to Apo-Proteins
$\gamma_{23} = \gamma_{25} = \gamma_{27} = \gamma_{29} = \gamma_{31} = \gamma_{33}$	Rate constant	$10^{-3} - 10^{2}$ 7 samples uniformly distributed in Log space	Recovery of glutathionylated proteins
$\gamma_{37} = \gamma_{38}$	Rate constant	$10^{-3} - 10^{2}$ 7 samples uniformly distributed in Log space	Export of ISC to the cytoplasm
γ_{41}	Rate constant	$0, 0.1$	Depletion of Yfh1
γ_{42}	Rate constant	$0, 0.1$	Depletion of Arh1_Yah1
γ_{43}	Rate constant	$0, 0.1$	Depletion of Ssq1 and Jac1
γ_{44}	Rate constant	$0, 0.1$	Depletion of Nfs1
γ_{45}	Rate constant	$0, 0.1$	Depletion of Grx5
$f_{33} = f_{53}$	Kinetic order	$0, 1, 2$	Assembly of ISC in scaffold
$f_{35} = f_{55}$	Kinetic order	$0, 1, 2$	Assembly of ISC in scaffold
f_{72}	Kinetic order	$0, 1, 2$	Correct folding of scaffolds
$f_{93}=f_{103}=f_{113}=f_{123}=f_{133}$	Kinetic order	$0, 1, 2$	Transfer of ISC to apo-proteins
$f_{94} = f_{104} = f_{114} = f_{124} = f_{134}$	Kinetic order	$0, 1, 2$	Transfer of ISC to apo-proteins
$f_{42} = f_{62}$	Kinetic order	$0, -1$	Stability of ISC in scaffolds
$f_{152} = f_{172} = f_{362}$	Kinetic order	$0, 1, 2$	*In situ* Repair of damaged clusters
$f_{153} = f_{173} = f_{363}$	Kinetic order	$0, 1, 2$	*In situ* Repair of damaged clusters
$f_{154} = f_{174} = f_{364}$	Kinetic order	$0, 1, 2$	*In situ* Repair of damaged clusters
$f_{155} = f_{175} = f_{365}$	Kinetic order	$0, 1, 2$	*In situ* Repair of damaged clusters
f_{232}	Kinetic order	$0, 1$	Deglutathionylation of scaffold
f_{252}	Kinetic order	$0, 1$	Deglutathionylation of P1
f_{272}	Kinetic order	$0, 1$	Deglutathionylation of P2
f_{292}	Kinetic order	$0, 1$	Deglutathionylation of Arh_1_Yah_1
f_{312}	Kinetic order	$0, 1$	Recovery of dead-end complex
f_{332}	Kinetic order	$0, 1$	Deglutathionylation of Nfs1

References

1. Camadro JM, Labbe P: **Purification and properties of ferrochelatase from the yeast Saccharomyces cerevisiae. Evidence for a precursor form of the protein**. *JBiolChem* 1988, **263**(24):11675-11682.
2. Ferreira GC, Franco R, Lloyd SG, Moura I, Moura JJ, Huynh BH: **Structure and function of ferrochelatase**. *JBioenergBiomembr* 1995, **27**(2):221-229.
3. Isaya G, O'Neill HA, Gakh O, Park S, Mantcheva R, Mooney SM: **Functional studies of frataxin**. *Acta PaediatrSuppl* 2004, **93**(445):68-71.
4. Park S, Gakh O, O'Neill HA, Mangravita A, Nichol H, Ferreira GC, Isaya G: **Yeast frataxin sequentially chaperones and stores iron by coupling protein assembly with iron oxidation**. *JBiolChem* 2003, **278**(33):31340-31351.
5. Gerber J, Muhlenhoff U, Lill R: **An interaction between frataxin and Isu1/Nfs1 that is crucial for Fe/S cluster synthesis on Isu1**. *EMBO Rep* 2003, **4**(9):906-911.
6. Layer G, Ollagnier de CS, Sanakis Y, Fontecave M: **Iron-sulfur cluster biosynthesis: Characterization of Escherichia coli cyay as an iron donor for the assembly of [2Fe-2S] clusters in the scaffold ISUC**. *JBiolChem* 2006.
7. Dutkiewicz R, Marszalek J, Schilke B, Craig EA, Lill R, Muhlenhoff U: **The Hsp70 chaperone Ssq1p is dispensable for iron-sulfur cluster formation on the scaffold protein Isu1p**. *JBiolChem* 2006, **281**(12):7801-7808.
8. Agar JN, Krebs C, Frazzon J, Huynh BH, Dean DR, Johnson MK: **IscU as a scaffold for iron-sulfur cluster biosynthesis: sequential assembly of [2Fe-2S] and [4Fe-4S] clusters in IscU**. *Biochemistry* 2000, **39**(27):7856-7862.
9. Adam AC, Bornhovd C, Prokisch H, Neupert W, Hell K: **The Nfs1 interacting protein Isd11 has an essential role in Fe/S cluster biogenesis in mitochondria**. *EMBO J* 2006, **25**(1):174-183.
10. Li J, Kogan M, Knight SA, Pain D, Dancis A: **Yeast mitochondrial protein, Nfs1p, coordinately regulates iron-sulfur cluster proteins, cellular iron uptake, and iron distribution**. *JBiolChem* 1999, **274**(46):33025-33034.
11. Wiedemann N, Urzica E, Guiard B, Muller H, Lohaus C, Meyer HE, Ryan MT, Meisinger C, Muhlenhoff U, Lill R *et al*: **Essential role of Isd11 in mitochondrial iron-sulfur cluster synthesis on Isu scaffold proteins**. *EMBO J* 2006, **25**(1):184-195.
12. Bonomi F, Iametti S, Ta D, Vickery LE: **Multiple turnover transfer of [2Fe2S] clusters by the iron-sulfur cluster assembly scaffold proteins IscU and IscA**. *JBiolChem* 2005, **280**(33):29513-29518.
13. Ding B, Smith ES, Ding H: **Mobilization of the iron centre in IscA for the iron-sulphur cluster assembly in IscU**. *BiochemJ* 2005, **389**(Pt 3):797-802.
14. Ding H, Harrison K, Lu J: **Thioredoxin reductase system mediates iron binding in IscA and iron delivery for the iron-sulfur cluster assembly in IscU**. *J Biol Chem* 2005, **280**(34):30432-30437.
15. Yang J, Bitoun JP, Ding H: **Interplay of IscA and IscU in Biogenesis of Iron-Sulfur Clusters**. *J Biol Chem* 2006, **281**(38):27956-27963.
16. Berndt C, Lillig CH, Wollenberg M, Bill E, Mansilla MC, de MD, Seidler A, Schwenn JD: **Characterization and reconstitution of a 4Fe-4S adenylyl sulfate/phosphoadenylyl sulfate reductase from Bacillus subtilis**. *JBiolChem* 2004, **279**(9):7850-7855.

17. Johnson DC, Dean DR, Smith AD, Johnson MK: **Structure, function, and formation of biological iron-sulfur clusters**. *AnnuRevBiochem* 2005, **74**:247-281.
18. Nishio K, Nakai M: **Transfer of iron-sulfur cluster from NifU to apoferredoxin**. *JBiolChem* 2000, **275**(30):22615-22618.
19. Wollenberg M, Berndt C, Bill E, Schwenn JD, Seidler A: **A dimer of the FeS cluster biosynthesis protein IscA from cyanobacteria binds a [2Fe2S] cluster between two protomers and transfers it to [2Fe2S] and [4Fe4S] apo proteins**. *EurJBiochem* 2003, **270**(8):1662-1671.
20. Yoon T, Cowan JA: **Iron-sulfur cluster biosynthesis. Characterization of frataxin as an iron donor for assembly of [2Fe-2S] clusters in ISU-type proteins**. *JAmChemSoc* 2003, **125**(20):6078-6084.
21. Yoon T, Cowan JA: **Frataxin-mediated iron delivery to ferrochelatase in the final step of heme biosynthesis**. *JBiolChem* 2004, **279**(25):25943-25946.
22. Kiley PJ, Beinert H: **The role of Fe-S proteins in sensing and regulation in bacteria**. *CurrOpinMicrobiol* 2003, **6**(2):181-185.
23. Agar JN, Yuvaniyama P, Jack RF, Cash VL, Smith AD, Dean DR, Johnson MK: **Modular organization and identification of a mononuclear iron-binding site within the NifU protein**. *JBiolInorgChem* 2000, **5**(2):167-177.
24. Mettert EL, Kiley PJ: **ClpXP-dependent proteolysis of FNR upon loss of its O2-sensing [4Fe-4S] cluster**. *JMolBiol* 2005, **354**(2):220-232.
25. Sutton VR, Mettert EL, Beinert H, Kiley PJ: **Kinetic analysis of the oxidative conversion of the [4Fe-4S]2+ cluster of FNR to a [2Fe-2S]2+ Cluster**. *JBacteriol* 2004, **186**(23):8018-8025.
26. Yang W, Rogers PA, Ding H: **Repair of nitric oxide-modified ferredoxin [2Fe-2S] cluster by cysteine desulfurase (IscS)**. *JBiolChem* 2002, **277**(15):12868-12873.
27. Kispal G, Csere P, Prohl C, Lill R: **The mitochondrial proteins Atm1p and Nfs1p are essential for biogenesis of cytosolic Fe/S proteins**. *EMBO J* 1999, **18**(14):3981-3989.
28. Lange H, Lisowsky T, Gerber J, Muhlenhoff U, Kispal G, Lill R: **An essential function of the mitochondrial sulfhydryl oxidase Erv1p/ALR in the maturation of cytosolic Fe/S proteins**. *EMBO Rep* 2001, **2**(8):715-720.
29. Tamarit J, Belli G, Cabiscol E, Herrero E, Ros J: **Biochemical characterization of yeast mitochondrial Grx5 monothiol glutaredoxin**. *JBiolChem* 2003, **278**(28):25745-25751.
30. Kato S, Mihara H, Kurihara T, Takahashi Y, Tokumoto U, Yoshimura T, Esaki N: **Cys-328 of IscS and Cys-63 of IscU are the sites of disulfide bridge formation in a covalently bound IscS/IscU complex: implications for the mechanism of iron-sulfur cluster assembly**. *ProcNatlAcadSciUS A* 2002, **99**(9):5948-5952.
31. Nuth M, Yoon T, Cowan JA: **Iron-sulfur cluster biosynthesis: characterization of iron nucleation sites for assembly of the [2Fe-2S]2+ cluster core in IscU proteins**. *JAmChemSoc* 2002, **124**(30):8774-8775.
32. Smith AD, Frazzon J, Dean DR, Johnson MK: **Role of conserved cysteines in mediating sulfur transfer from IscS to IscU**. *FEBS Lett* 2005, **579**(23):5236-5240.
33. Andrew AJ, Dutkiewicz R, Knieszner H, Craig EA, Marszalek J: **Characterization of the interaction between the J-protein Jac1 and Isu1, the scaffold for Fe-S cluster biogenesis**. *JBiolChem* 2006.

34. Dutkiewicz R, Schilke B, Cheng S, Knieszner H, Craig EA, Marszalek J: **Sequence-specific interaction between mitochondrial Fe-S scaffold protein Isu and Hsp70 Ssq1 is essential for their in vivo function**. *JBiolChem* 2004, **279**(28):29167-29174.
35. Dutkiewicz R, Schilke B, Knieszner H, Walter W, Craig EA, Marszalek J: **Ssq1, a mitochondrial Hsp70 involved in iron-sulfur (Fe/S) center biogenesis. Similarities to and differences from its bacterial counterpart**. *JBiolChem* 2003, **278**(32):29719-29727.
36. Knieszner H, Schilke B, Dutkiewicz R, D'Silva P, Cheng S, Ohlson M, Craig EA, Marszalek J: **Compensation for a defective interaction of the hsp70 ssq1 with the mitochondrial Fe-S cluster scaffold isu**. *JBiolChem* 2005, **280**(32):28966-28972.
37. Schmidt S, Strub A, Rottgers K, Zufall N, Voos W: **The two mitochondrial heat shock proteins 70, Ssc1 and Ssq1, compete for the cochaperone Mge1**. *JMolBiol* 2001, **313**(1):13-26.
38. Voisine C, Cheng YC, Ohlson M, Schilke B, Hoff K, Beinert H, Marszalek J, Craig EA: **Jac1, a mitochondrial J-type chaperone, is involved in the biogenesis of Fe/S clusters in Saccharomyces cerevisiae**. *ProcNatlAcadSciUS A* 2001, **98**(4):1483-1488.
39. Voisine C, Schilke B, Ohlson M, Beinert H, Marszalek J, Craig EA: **Role of the mitochondrial Hsp70s, Ssc1 and Ssq1, in the maturation of Yfh1**. *MolCell Biol* 2000, **20**(10):3677-3684.
40. Silberg JJ, Hoff KG, Tapley TL, Vickery LE: **The Fe/S assembly protein IscU behaves as a substrate for the molecular chaperone Hsc66 from Escherichia coli**. *JBiolChem* 2001, **276**(3):1696-1700.
41. Savageau MA: **Biochemical systems analysis. I. Some mathematical properties of the rate law for the component enzymatic reactions**. *J Theor Biol* 1969, **25**(3):365-369.
42. Savageau MA: **Biochemical systems analysis. II. The steady-state solutions for an n-pool system using a power-law approximation**. *J Theor Biol* 1969, **25**(3):370-379.
43. Sorribas A, Savageau MA: **Strategies for representing metabolic pathways within biochemical systems theory: reversible pathways**. *Math Biosci* 1989, **94**(2):239-269.
44. Voit EO, Savageau MA: **Accuracy of alternative representations for integrated biochemical systems**. *Biochemistry* 1987, **26**(21):6869-6880.
